# Physical model experiment on the influence of water depth on the underwater pipeline surface impacted by landslide surge

**DOI:** 10.1038/s41598-021-98324-x

**Published:** 2021-09-29

**Authors:** Hong Ji, Weikang Liu, Ke Yang, Juncheng Jiang, Zhixiang Xing, Yaxin Wang, Shuhua Zhao

**Affiliations:** 1 School of Environment and Safety Engineering, Changzhou University, Changzhou, 213164 Jiangsu China; 2 Jiangsu Key Laboratory of Oil and Gas Storage and Transportation Technology, Changzhou University, Changzhou, 213016 Jiangsu China; 3 School of Petroleum Engineering, Changzhou University, Changzhou, 213016 Jiangsu China

**Keywords:** Natural hazards, Ocean sciences

## Abstract

A physical model experiment of flume block landslide was used to study the influence of landslide surge impact on underwater pipeline surface under different water depths. The influence of surge impact pressure on pipelines with different water depths and the impact pressure of surge at different angles of underwater pipelines wall were analyzed. And the relationship between the maximum impact pressure of underwater pipelines and the depth of water was obtained. The results indicated that with the decrease of the water depths, the maximum impact pressure at the wall of the underwater pipeline increases approximately linearly, and the slider is easier to form higher first wave height. The maximum impact pressure of the upper surface of the pipeline wall is greater than that of the lower surface of the pipeline wall under the same working conditions. It is also found that the smaller the depth of water, the larger the maximum pressure and average pressure at the measuring point would be and the greater the pressure fluctuation becomes when slider volume and landslide water inlet angle and speed remain the same.

## Introduction

Surge is a secondary disaster caused by high speed water entry of landslide, which not only poses a threat to the safety of reservoir or watercourse, but also causes casualties and property losses, as well as more disasters such as flood and overflow dam easily^1–3^. On October 9, 1963, the Banks of the Vaiont Reservoir in Italy suddenly slipped a super-giant landslide with a volume of 240 million m^3^, causing a huge wave of about 175 m height, causing surrounding towns to be destroyed by flood, leading more than 2,000 people casualties and causing the most destructive landslide accident in history^4^. On July 13, 2003, a huge landslide of about 24 million m^3^ occurred in ZiGui County along the QingGan River, a tributary of the Yangtze River. The huge wave, which was more than 20 m height, overturning 22 boats, and causing 14 deaths and 10 missing, caused economic losses of about 80 million yuan^5,6^. Due to the frequent occurrence of severe weather and the construction of water conservancy projects, the surrounding geological conditions are changed, which leads to the slide of the reservoir bank and other rock masses, resulting in the landslide surge accident.

At present, relevant researchers at home and abroad use physical model test methods to study the surge height, climbing slope and the change of the surge shape after the landslide enters the water. Hu et al.^7^ studied the failure mode and deformation characteristics of the landslides in the Three Gorges Reservoir region. Xu et al.^8^ analyzed the destruction mechanism of landslide dams due to a surge and the influence of the landslide height, the contact area, and the distance between the dam site and the entry point for a dam break. Huang et al.^1^ established a source model of landslide-induced waves in shallow water and calculated wave propagation and run-up. Li et al.^9^ proposed the height of the initial surge, empirical formula of soil-landslide surge and variation of the progressive surge. Yavari-Ramshe et al.^10^ analyzed the impulse wave characteristics and deformation of landslide and studied the influence of landslide deformation on water surface fluctuation. Huang et al.^11^ constructed a large-scale physical Froude-similar model of producing impulse waves based on the Chinese Gongjiafang landslide. Peng et al.^12^ established large-scale wave flume tests to study the erosion failure modes of dam body caused by surges and the variation in the pore water pressure in the dam body. Schnyder et al.^13^ used numerical simulation methods to reconstruct landslides modeled with a velocity of 20 ms^−1^ and a few cubic kilometers of volume (1.41–5.53 km^3^) and simulate the influence of submarine landslides impact. Yue et al.^14^ revealed the initial formation characteristic and the attenuation law of landslide-generated waves. Carvalho et al.^15^ analyzed several processes, such as sliding block into reservoir, generation and propagation of impact surge, and influence of impact surge on downstream, through experimental study. Yin et al.^16^ studied the influence of comprehensive factors on the shape change of landslide surge by indoor large-scale physical model experiment, and proposed the calculation formula of landslide surge. Heller and Valentin^17^ found that the wave height of surge was generally 5/4 times of the wave amplitude, and the wave velocity was generally close to the theoretical wave velocity of isolated wave through 211 physical similarity tests. Tian et al.^18^ studied the impact of landslide volume, the initial wave height and the reservoir water depth on bridge pier through physical model of the surge. Wang et al.^19^ studied the effects of landslide generated impulse waves on ship impact force for pile wharf. Bai et al.^20^ studied characteristics of impact force brought by landslide surge on the pile and worked out a formula for calculating the extreme value of dynamic hydraulic pressure on the pile. Zakeri et al.^21–23^ simulated the impact of the debris flow from the submarine landslide on the submarine pipeline by the flume experiment and numerical analysis, and developed a method for estimating the drag force exerted when the debris flow impacts the pipeline. Min et al.^24^ proposed a new deterministic landslide risk assessment method based on machine learning algorithms. Guo et al.^25^ proposed a quantitative procedure method analyze and assess the landslide risks to building and lives by considering the Outang landslide as an example.

There are many factors affecting the landslide surge impact force on the underwater pipeline, including landslide volume, depth of water, morphology of landslide front, and velocity and angle of entry of landslide, etc^26,27^. Xu^28,29^ studied the influence of landslide body shape, morphology of landslide front, landslide volume, the water surface width and friction factor of slide surface on the surge. Tang et al.^30^ examined the effects of the mass ratio, hill slope angle and grain size on impulse waves generated by combined block-granular landslides. Li and Sun^31,32^ studied the influences of particle space, initial falling velocity water depth and slider width etc. on the surge wave height and impact pressure on dam surface were analyzed.

Previous studies mainly focused on the morphological characteristics, propagation attenuation and wave height of landslide surge, while the impact pressure of surge waves was rarely studied. Among the reasons for the failure of underwater oil and gas pipelines, the impact of landslide surges is one of the main influencing factors, and there is a lack of experimental and theoretical research on the impact of landslide surges on underwater oil and gas pipelines. Therefore, through physical model experiment, the influence of impact force of surge generated by the slider on the surface of underwater pipelines at different water depths is studied, and the law of the impact pressure of underwater pipeline varying with the depth of water is obtained in this paper.

## Experimental model

**Figure 1:**
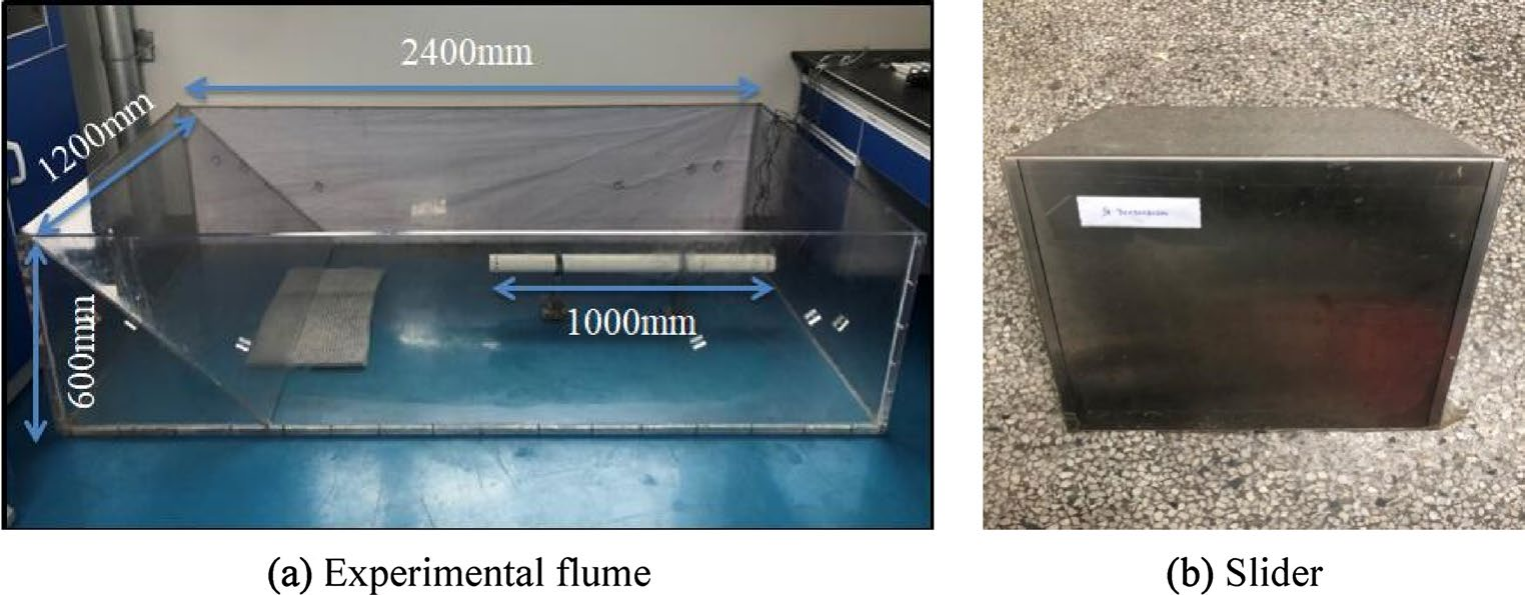
Flume device and slide block (**a**) experimental flume, (**b**) slider.

**Figure 2:**
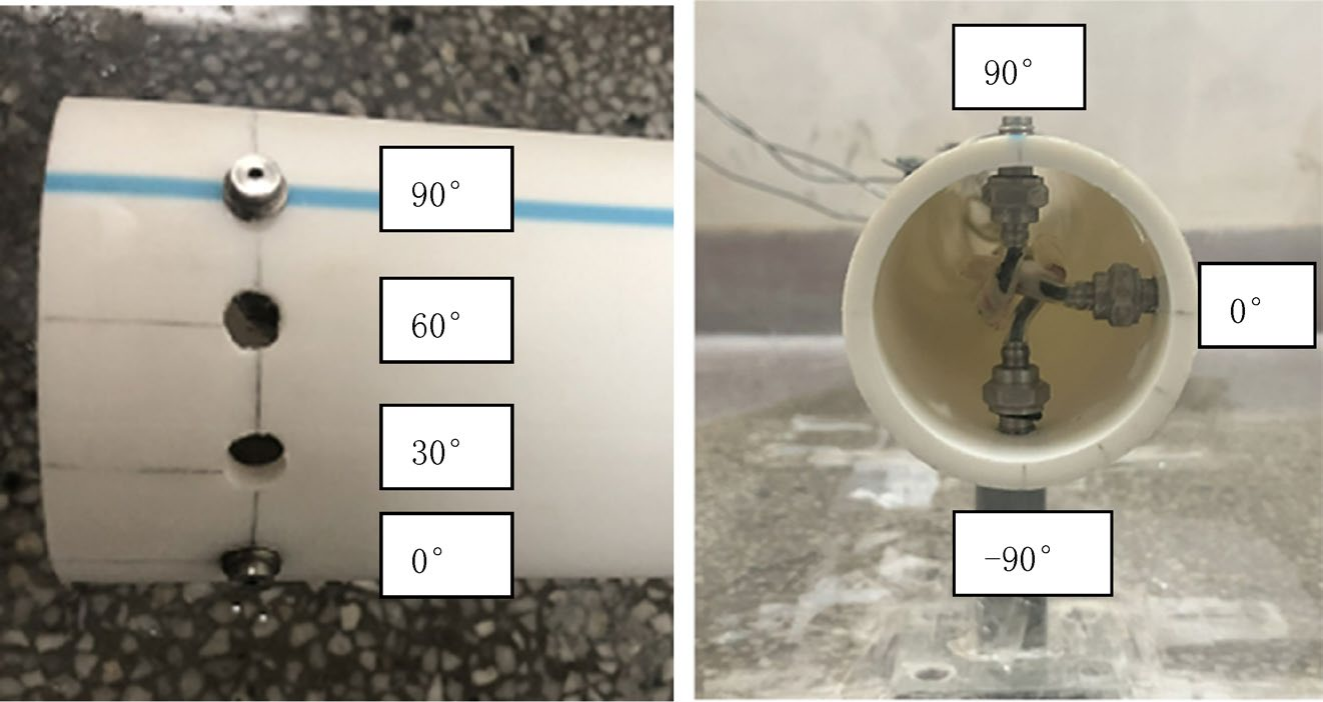
Position of pressure probe.

**Figure 3:**
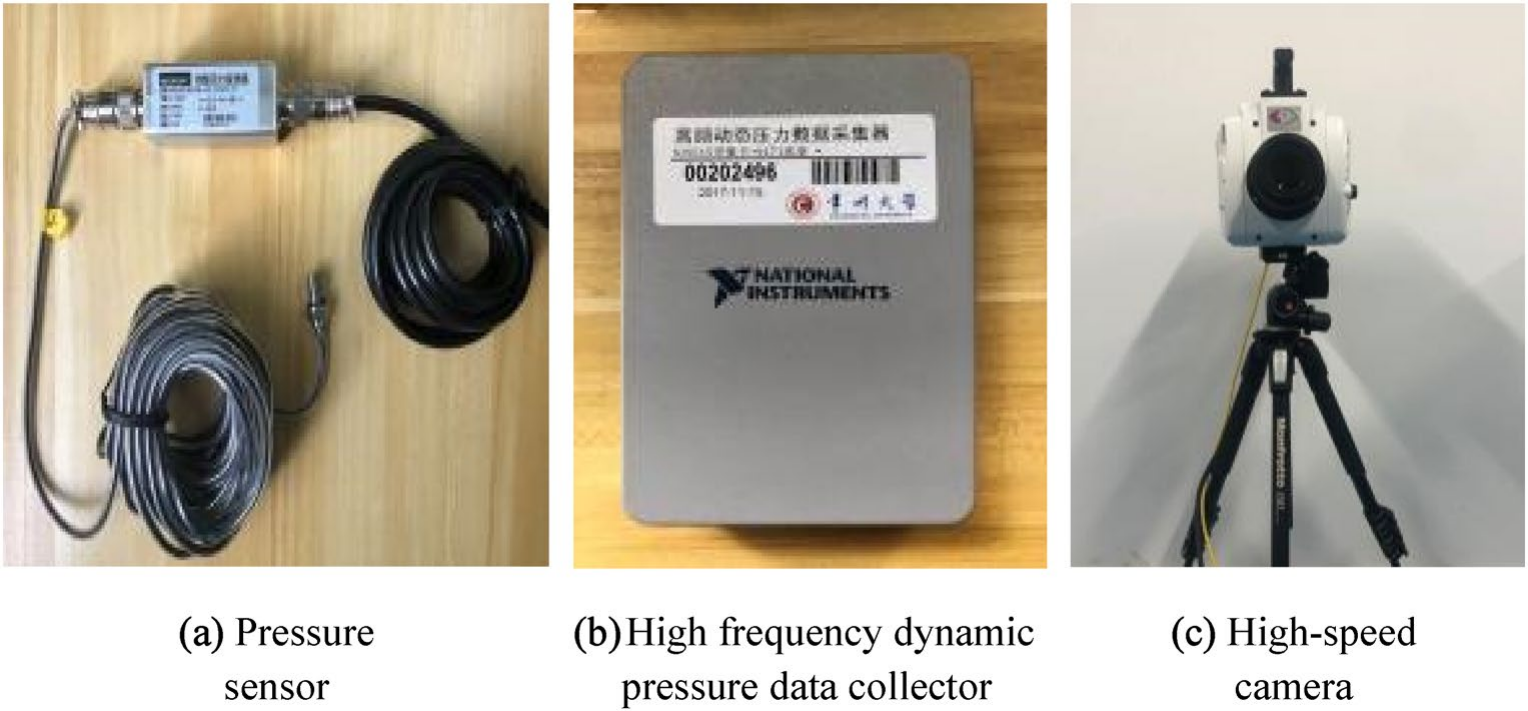
Experimental facilities (**a**) pressure sensor, (**b**) high frequency dynamic pressure data collector, (**c**) high-speed camera.

The experiment was conducted in an artificial flume made of Polymethyl Methacrylate (PMMA) material to study the impact of surge generated by landslide in different water depths on the surface of underwater pipeline. The experiment flume is a length of 2.4 m, a width of 1.2 m, and a height of 0.6 m (Fig. 1a). The underwater pipeline is PP-R pipeline, with a length of 1 m, an outside diameter of 0.063 m and a pipe thickness of 0.0058 m. Three openings with a diameter of 0.008 m are set 0.02 m away from the left edge of the pipeline. The pipeline is fixed at the bottom of the flume through the support frame. The sliding block used in the experiment is rigid slide block, the shape and size are shown in Fig. 1b. As shown in Fig. 2, the center of the cross-section circle of the pipe is taken as the origin of the plane coordinate system, and the top, bottom and right ends of the pipe are defined as the monitoring points of 90°, -90° and 0° of the pipe surface respectively. And a pressure probe is installed at each monitoring point. The MD-MT200 miniature pressure transmitter is used for pressure transmission with a measuring range of 0-20 kPa and accuracy of 0.5%. The probes of the three miniature pressure transmitters (Fig. 3a) are connected with the three openings on the left side of the pipe through threads. The sampling frequency of the high frequency dynamic pressure data collector (Fig. 3b) is set to 10,000 Hz, and the sensor sensitivity is 500mv/KPa. Phantom high-speed camera (Fig. 3c) is used for image acquisition, with a resolution of 1280 × 800 and a frame number of 10,000 frames per second.

In this paper, the control variable method is adopted to design the experiment. Each group of experiments was conducted three times, and the average value of the obtained data was taken to avoid the contingency of the experiment. The parameter values are shown in Table 1.

**Table 1:** Parameter value.

Case	Water depth *h* (m)	Angle of entry of landslide *α* (°)	Relative height of the slider and the water surface *h* (m)	Landslide volume *ν* (m^3^)
1	0.30	45	0.15	0.006
2	0.32	45	0.15	0.006
3	0.34	45	0.15	0.006
4	0.36	45	0.15	0.006
5	0.38	45	0.15	0.006
6	0.40	45	0.15	0.006

## Results and discussions

### Surge variation at different depths

**Figure 4:**
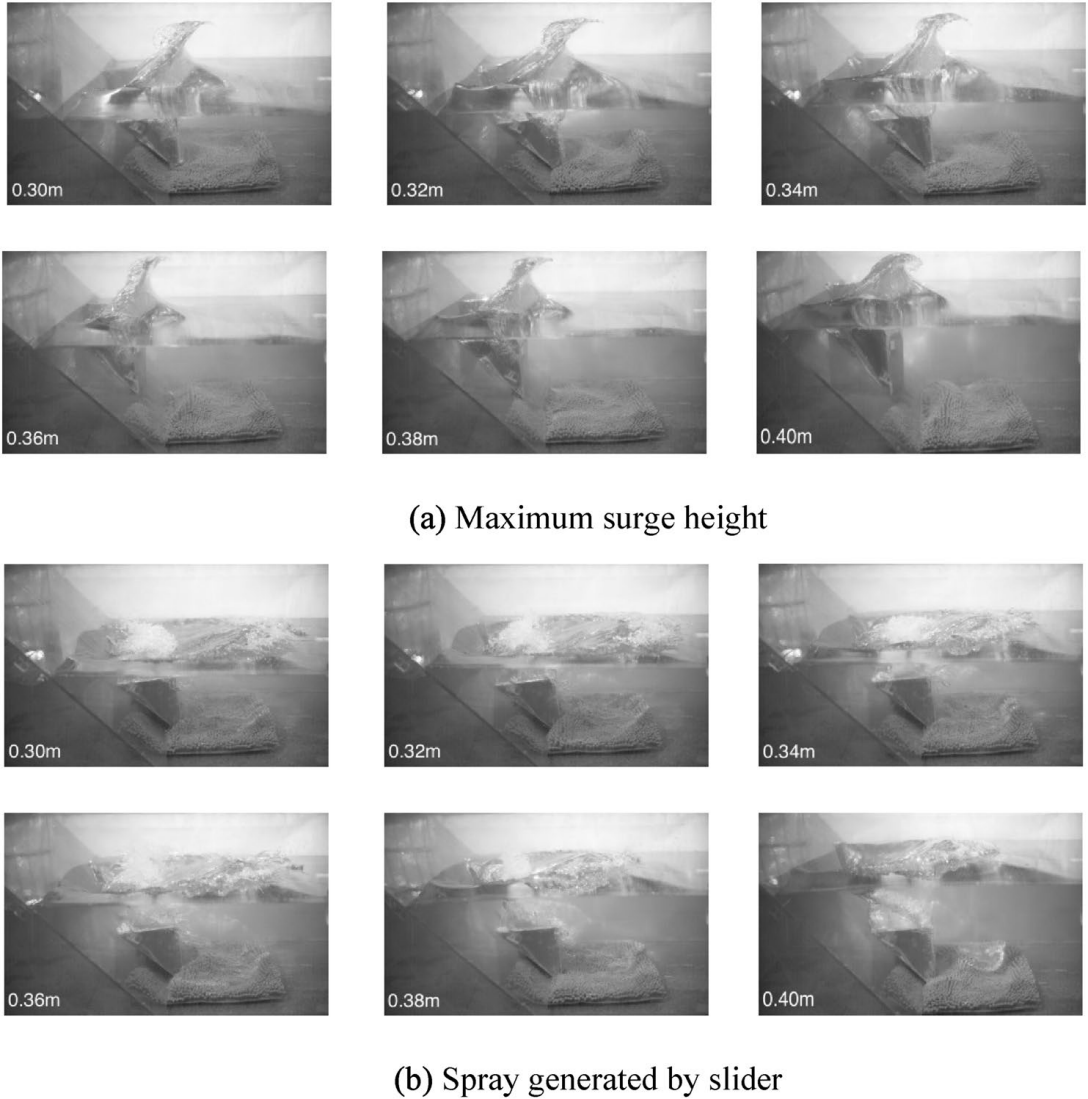
Surge form (**a**) maximum surge height (**b**) spray generated by slider.

As shown in Fig. 4, the shape diagram of surge formed by sliding block entering water under 6 different water depth conditions in shallow water environment. Figure 4a shows the maximum surge height formed by the slider when it slides into the water of different depths, and Fig. 4b shows the morphology of it when the maximum surge falls onto the water surface. After the sliding block enters the water, the water body is impacted and squeezed by the sliding block, and the water body rises rapidly. When the maximum surging wave is reached, a roll-breaking wave moving forward is formed. With the air brought into the water body by the sliding block and roll-breaking wave, the water surface appears splashing. As the air brought into the water by the sliders and the coil breakers rises and rises to the surface, making the water surface appear splashing. It can be seen from the Fig. 4a that as the depth of water increases, the maximum surge height formed by the slider entering the water is decreasing; when the maximum surge height is formed, the positions of sliders of different water depth are also different. In the water depth of 0.30–0.34 m, the sliders have already slid into the bottom of flume, while in the water depth of 0.36–0.40 m, the sliders have not yet slid into the bottom of flume. In the Fig. 4b, it can be observed that the shallower the water, the larger and wider the spray formed as the maximum surge falls onto the water surface; the tumbling spray appears in the cavity at the tail of the slider, and the shallower the water, the more intense the splash.

In the case of the same velocity and angle of entry of landslide and landslide volume, as the water depth increases, the energy transferred from the slide block to the water body increases, and the maximum wave height of the first wave produced by the horizontal extrusion decreases, indicating that in the case of shallow water, the slide block is more likely to form a higher first wave height. However, with the surge spreading, the stable wave height of the surge tends to be the same under different water depths.

**Figure 5:**
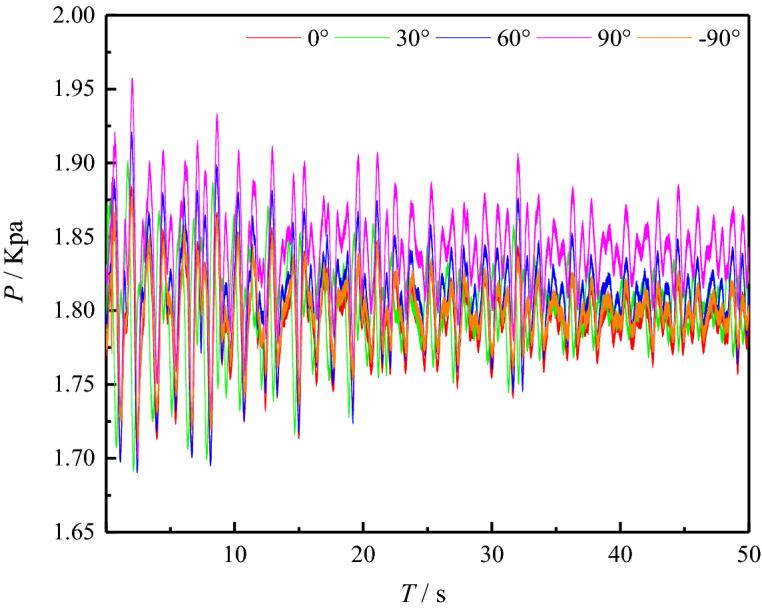
Pressure fluctuation curves at various positions of underwater pipeline wall.

Figure 5 shows the experimental data of working condition 6, from which the measured value of surge impact pressure at different positions on the underwater pipeline wall can be obtained. On the whole, as time goes by, the pressure value is constantly fluctuating and changing, and as the surge gradually attenuates, the wave crest gradually decreases, and the pressure value shows a gradual decrease trend. At the beginning, the slide block continuously slides into the water body, and the volume of the squeezed water body increases. Therefore, the surge impact pressure at each measuring point on the wall of the underwater pipeline is increasing over a period of time. It can also be observed that the variation trend of the pressure value at different locations of the underwater pipeline is roughly the same, but the pressure fluctuation degree and the maximum pressure value at different positions of the pipeline are obviously different, among which the maximum pressure of the measurement point of 90° is 1.954 kPa at 2.067 s.

### Effect of surge on the surface of underwater pipeline

**Figure 6:**
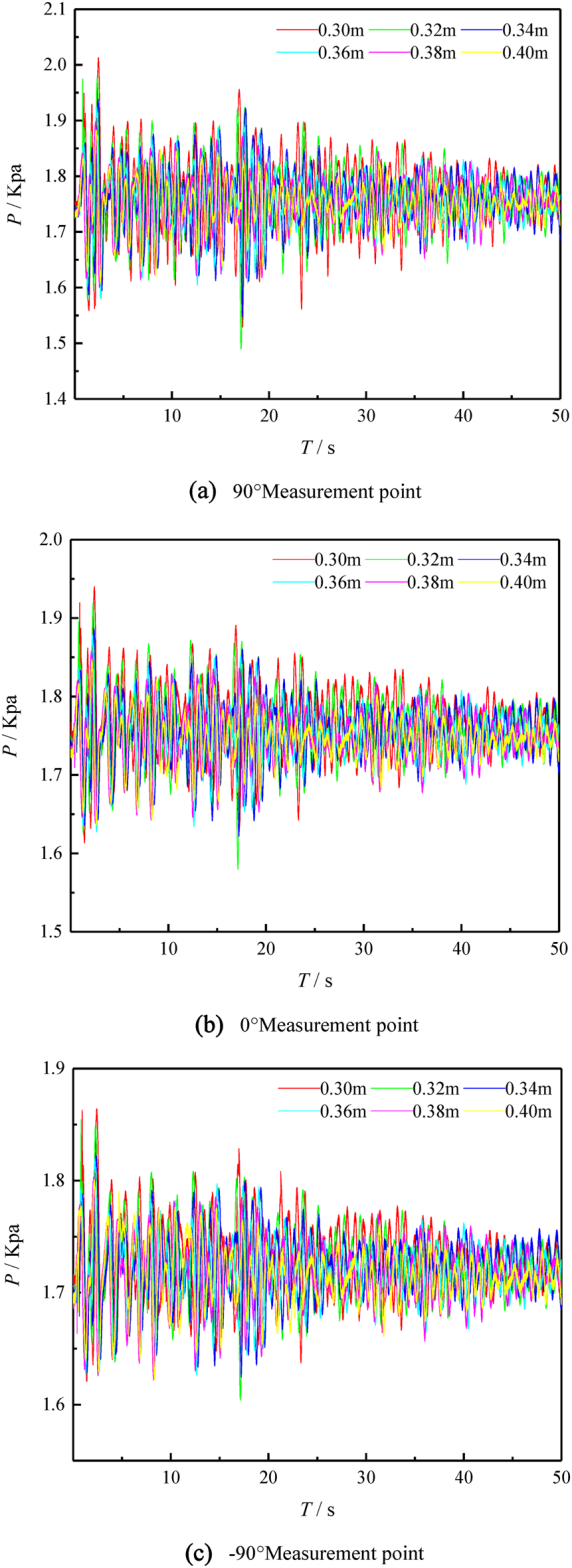
Pressure fluctuation curves at 90°, 0° and − 90° measuring points (**a**) 90° measurement point (**b**) 0° measurement point (**c**) − 90° measurement point.

Figure 6 shows the curve of pressure fluctuation when the surge formed by the sliding block entering the water acts on the three measuring points of the underwater pipeline 90°, 0° and -90° under the working conditions of 1–6. It can be obtained from the figure that, with the passage of time and the attenuation of the surge, the surge impact pressure at the three measuring points presents a general trend of decreasing. The shallower the water, the surge impact pressure at the three measuring points of the underwater pipeline is greater. At the beginning, as the slide block continuously slides into the water body, energy is transferred from the slide block to the water body, and the volume of the water body squeezed by the slide block increases, generating the first surge. Therefore, the surge impact pressure on the wall of the underwater pipeline keeps increasing over a period of time, and when it reaches a certain peak, the pressure value decreases. During the sliding process of the slide block, part of the air is brought into the water body, and the air overflows from the water body, making the elevation of the water surface in front of the slope began to rise. After reaching a certain elevation, the elevation of the water surface in front of the slope began to fall. In the process of surge forward, the water falling back from the slope surface also begins to form more regular surge. The forward movement, reflection and superposition of the surge constantly impact the pipeline, and the pressure value fluctuates constantly.

By comparing the three measuring points of the underwater pipeline, it is found that the maximum pressure at the measuring point of 90° is the largest, and the degree of pressure fluctuation is the largest. The maximum pressure at the measuring point of − 90° is the lowest, and the degree of pressure fluctuation is the lowest. Under the conditions of the same angle and velocity of entry of the landslide and the same volume of the slide block, the shallower the water, the greater the surge impact pressure on the underwater pipeline and the greater the degree of pressure fluctuation. Through the comparison of experimental data, the maximum surge pressure value and the maximum pressure fluctuation degree of underwater pipeline are at water depth h = 0.30 m, angle of entry of landslide = 45°, relative height of the slider and the water surface h = 0.15 m, the landslide volume = 0.006 m^3^ and 90° measurement point of underwater pipeline.

The experiment in each group was conducted three times, and the pressure data shown in Table 2 were obtained by averaging the data of the three experiments. Under the conditions of the same angle and velocity of entry of the landslide and the same volume of the slide block, the shallower the water, the larger the maximum pressure, the average pressure and the standard deviation at the three measuring points of the underwater pipeline. When all working conditions are the same, the maximum pressure, the average pressure and the standard deviation of the underwater pipeline are the largest at the 90°measuring point, while the maximum pressure, the average pressure and the standard deviation of the underwater pipeline are the smallest at the − 90°measuring point.

**Table 2:** Pressure data at 90°, 0° and − 90°measuring points of underwater pipelines in different water depths.

Water depth h (m)	Maximum pressure P (Kpa)			Average pressure P (Kpa)			Standard deviation		
	90°	0°	− 90°	90°	0°	− 90°	90°	0°	− 90°
0.30	2.01302	1.94030	1.86416	1.76797	1.76766	1.72953	0.06435	0.04265	0.03241
0.32	1.97996	1.91925	1.85442	1.76211	1.76208	1.72460	0.06019	0.04187	0.03231
0.34	1.93799	1.88619	1.82239	1.75974	1.75598	1.72260	0.05387	0.03750	0.02904
0.36	1.92488	1.87854	1.81987	1.75530	1.75194	1.71876	0.04949	0.03629	0.02896
0.38	1.89847	1.85749	1.80333	1.75450	1.74964	1.71582	0.04503	0.03422	0.02805
0.40	1.88371	1.84776	1.80101	1.75169	1.74620	1.71489	0.03804	0.03102	0.02555

The experimental data showed that when the water depth is the smallest, the impact pressure of landslide surge on the pipe wall closer to the water surface was the largest, and the pressure fluctuation was the largest; while the water depth was the largest, the impact pressure of landslide surge on the pipe wall closer to the water surface was the smallest, and the pressure fluctuation was the smallest. This indicates that the landslide surge has great influence on the pressure near the water surface, and the pressure fluctuates obviously.

### Relationship between maximum impact pressure and water depth

During the process of the slide block impacting the water body to form the surge, the impact pressure at each measuring point on the wall of the underwater pipeline reached the maximum pressure at a certain moment. The maximum pressure value impacted by surge is of great significance for the safe operation of pipeline. The data of the maximum impacted pressure at each measuring point was obtained through the experiments, and the relationship between the maximum impact pressure at different measuring points of the underwater pipeline and water depth was analyzed respectively.

**Figure 7:**
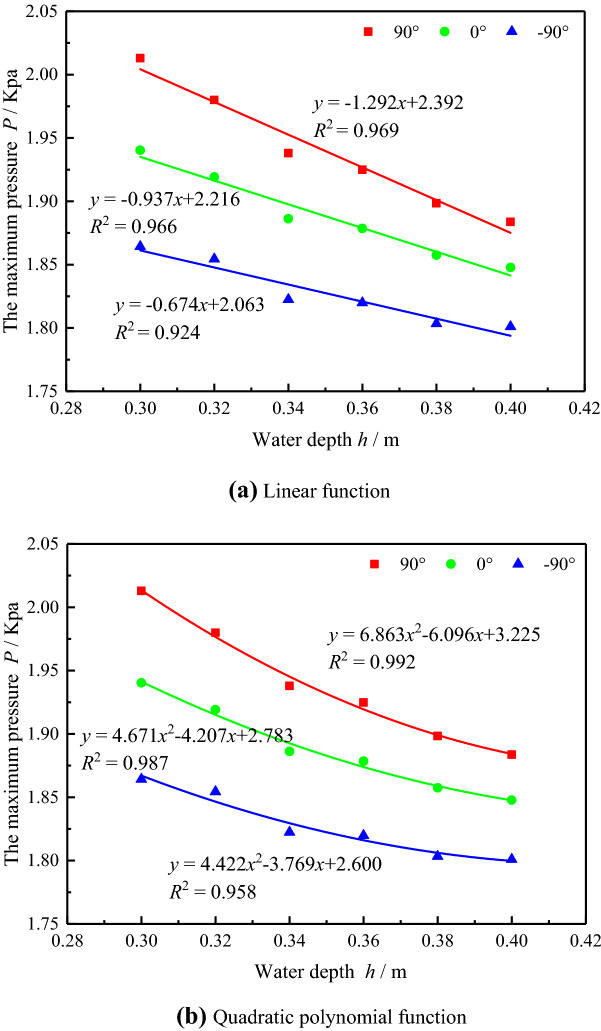
Relationship between surge impact pressure and water depth (**a**) linear function (**b**) quadratic polynomial function.

Linear function and quadratic polynomial function were respectively used to fit the relationship between water depth and the maximum surge impact pressure at the 90°, 0° and − 90°measuring points of underwater pipeline. It can be obtained from Fig. 7 that with the increase of water depth, the maximum surge impact pressure at the 90°, 0° and − 90° measuring points of the underwater pipeline presents a law of approximate linear decline. Formula (1), (2) and (3) were respectively the linear function fitting relations between the maximum surge impact pressure at the 90°, 0° and -90°measuring points of the underwater pipeline and water depth; formula (4), (5) and (6) were respectively the quadratic polynomial function fitting relations between the maximum surge impact pressure at the 90°, 0° and − 90°measuring points of the underwater pipeline and water depth. In Table 3, R^2^ is the fitting correlation coefficient. The closer R^2^ is to 1, the better the fitting correlation is. The correlation coefficient of linear function and quadratic polynomial function fitting are both close to 1, but the correlation of quadratic polynomial function fitting is better. Therefore, quadratic polynomial fitting function can provide reference for the prediction of impact pressure of underwater pipeline at different water depth.

1 $$y = - 1.292x + 2.3932$$

2 $$y = - 0.937x + 2.216$$

3 $${\text{y}} = - 0.674x + 2.063$$

4 $$y = 6.863x^{2} - 6.096x + 3.225$$

5 $$y = 4.671x^{2} - 4.207x + 2.783$$

6 $$y = 4.422x^{2} - 3.769x + 2.600$$

**Table 3:** Value of R^2^.

Measure point	Linear function	Quadratic polynomial function
90°	0.969	0.992
0°	0.966	0.987
− 90°	0.924	0.958

## Principle of similitude

### Similitude theory

The similitude principle is the theoretical basis of the model test, mainly including three similar theorems, which are the basis of the model design, data processing. Based on the similar theorems, various empirical formulas in engineering have been established. The first similitude theorem can be expressed that for a similar phenomenon, the similarity index is equal to 1. This theorem derives from the known fact, and also is the inevitable results of a similar phenomenon, illustrating the basic properties of similarity. The second similitude theorem can be expressed that physical equations describing natural phenomenon can be expressed as a functional relationship between similarity criteria numbers. The third similitude theorem can be expressed that for the same type of physical phenomenon, if the single value quantities are similar and the similarity criteria numbers being made of single value quantities are the same, the phenomenon is similar. Single value quantities refer to the physical quantities in single value conditions, which include geometric condition, boundary condition, and initial condition.

### Similitude experimental design

The liquid movement is carried out in a certain space and time, following the basic laws of fluid kinematics and dynamics. The physical quantities representing fluid moving process are divided into three kinds, which are the geometry of the flow field, the state of motion, and dynamic characteristics. According to the third similitude theorem, two similar flow systems must ensure geometric similitude, dynamic similitude, and kinematic similitude.

#### Geometric similitude

Geometric similitude requires the scale of the prototype and model to maintain a certain ratio. In more detail, suppose that a geometric body, its volume is $V$, the area is $S$, the length is $l$, and the angle is $\theta$, then the similar constant is expressed as follows.

7 $$C_{l} = \frac{{l_{p} }}{{l_{m} }},\;C_{\theta } = \frac{{\theta_{p} }}{{\theta_{m} }},\;C{}_{S} = \frac{{S_{p} }}{{S_{m} }} = \frac{{l_{p}^{2} }}{{l_{m}^{2} }},\;C_{V} = \frac{{V_{p} }}{{V_{m} }} = \frac{{l_{p}^{3} }}{{l_{m}^{3} }}$$

Among the submarine pipelines that have been laid, small-diameter pipes are 50.8–254 mm (2–10 in), medium-diameter pipes are 304.8–609 mm (12–24 in), and large-diameter pipes are 609.6–863.6 mm (24–34 in). According to specific application cases, the similarity coefficient can be determined. Take the large-diameter pipe as an example, the similarity ratio of the pipe diameter between the model and the prototype is 1:10. Then

$$C_{l} = \frac{{l_{p} }}{{l_{m} }} = 10,\;C{}_{S} = \frac{{S_{p} }}{{S_{m} }} = \frac{{l_{p}^{2} }}{{l_{m}^{2} }} = 10^{2} ,\;C_{V} = \frac{{V_{p} }}{{V_{m} }} = \frac{{l_{p}^{3} }}{{l_{m}^{3} }} = 10^{3} .$$

#### Dynamic similitude

Dynamic similarity refers that the forces of the different nature in the model and prototype system are in the same proportional relationship. In general, forces in fluid system include gravity, inertial force, viscous force, surface tension, the elastic force and so on. As long as the external forces playing a dominant role meet the similar conditions, the model test will be able to reflect the state of motion of the fluid. The principal force of the surge simulation is gravity. According to the Froude model law, Froude number (Fr) of the model and prototype being equal is the necessary and sufficient condition of gravity power being similar. Then

8 $$F_{rp} = \frac{{v_{p} }}{{\left( {g_{p} l_{p} } \right)^{1/2} }} = F_{rm} = \frac{{v_{m} }}{{\left( {g_{m} l_{m} } \right)^{1/2} }}.$$

The similar constant of density

$$C_{\rho } = \frac{{\rho_{p} }}{{\rho_{m} }} = 1.$$

The similar constant of gravity acceleration

$$C_{g} = \frac{{g_{p} }}{{g_{m} }} = 1.$$

The similar constant of quality

$$C_{m} = \frac{{m_{p} }}{{m_{m} }} = \frac{{\rho_{p} V_{p} }}{{\rho_{m} V_{m} }} = C_{\rho } C_{V} = C_{l}^{3} = 10^{3} .$$

The similar constant of gravity

$$C_{G} = C_{m} C_{g} = C_{l}^{3} = 10^{3} .$$

#### Kinematic similitude

Kinematic similitude refers to that the trace of the corresponding particle in model and prototype is geometrically similar, and the time of particle flowing corresponding segment is in the same proportion. The velocity field and the acceleration field of the model and prototype are similar. Suppose that $v_{p}$ is the flow velocity at a point in the prototype, and $v_{m}$ is the flow velocity at the corresponding point in the model, then the scale of flow velocity is as follows.

9 $$C_{v} = \frac{{v_{p} }}{{v_{m} }}.$$

According to the Eq. 9, the following results can be gained.

$$C_{v} = \frac{{v_{p} }}{{v_{m} }} = \left( {\frac{{l_{p} }}{{l_{m} }}} \right)^{1/2} = \sqrt {10}$$

$$C_{t} = \frac{{t_{p} }}{{t_{m} }} = \frac{{C_{l} }}{{C_{v} }} = 10$$

## Conclusion

Through the physical model experiment, the influence of landslide surge impact on the surface of underwater pipeline under different water depths was studied, the motion characteristics of the surge generated by the slide block entering into the water were analyzed. Furthermore, the relationship between the maximum impact pressure of the underwater pipeline and the water depth was obtained. The conclusions are as follows:

1. Under the condition of the same slide block volume and the same angle and velocity of entry of landslide, with the decrease of depth of water, the maximum impact dynamic pressure on the wall of the underwater pipeline presented an approximately linear increasing law. Within a certain range, the shallower the water, the greater the maximum pressure and the average pressure at each measuring point of the underwater pipeline, and the greater the degree of pressure fluctuation.
2. When all working conditions were the same, the maximum pressure, the average pressure and the standard deviation of the underwater pipeline were the largest at the 90° measuring point, and on the contrary, the minimum value was at the − 90° measuring point. This indicated that the impact strength of surges increased closer to the water surface.
3. According to the relation between the maximum surge impact pressure of underwater pipeline and water depth, as well as R^2^ value, it can be seen that the relation between the maximum impact pressure of the underwater pipeline and water depth fitted by quadratic polynomial function is better, which can provide reference for the prediction of impact pressure of underwater pipeline at different water depth.
4. Considering that gravity plays a major role in the impact of surge on the pipeline, the Froude similarity criterion is selected and the similarity coefficient is calculated. Among the submarine pipelines that have been laid, small-diameter pipes are 50.8–254 mm (2–10 in), medium-diameter pipes are 304.8–609 mm (12–24 in), and large-diameter pipes are 609.6–863.6 mm (24–34 in). According to specific application cases, the similarity coefficient can be determined. Take the large-diameter pipe as an example, the similarity ratio of the pipe diameter between the model and the prototype is 1:10.
